# Supernumerary nipple in a young woman

**DOI:** 10.11604/pamj.2019.34.36.17762

**Published:** 2019-09-17

**Authors:** Salma Salim, Badreddine Hassam

**Affiliations:** 1 Dermatology and Venereology Department, Ibn Sina University Hospital, Rabat, Morocco

**Keywords:** Supernumerary nipple, milk line, associated malforamtions and neoplasms

## Image in medicine

We report the case of a 24 year old woman, without significant past medical history, who consulted for an asymptomatic and pigmented lesion of the breast evolving from infancy. Dermatological examination showed a brown plaque localized on the milk line in the underside of the right breast, measuring 1 cm, firm in consistency, with a non infiltrated base. Dermoscopic examination revealed a peripheral pigmented regular network with a cleft-like appearance in a pigmented homogeneous central area. The rest of clinical examination was normal. Based on the clinical and dermoscopic findings a diagnosis of supernumerary nipple was established. The patient refused the removal of the nipple since it was asymptomatic and without any aesthetic concern. Supernumerary nipples are congenital malformations of nipples that arise in addition to the normal bilateral nipples. Their prevalence is between 0.22% and 5.6%. They most commonly arise along the milk lines, which extend from axillae to inguinal region bilaterally. However, they may also appear in other regions of the body and may be associated with underlying breast tissue. Supernumerary nipples may be associated with other abnormalities of the central nervous, gastrointestinal, respiratory, skeletal or cardiovascular systems and sometimes genetic and chromosomal abnormalities. Nevertheless, the most known is the association with genitourinary tract malformations and malignancies. Given these morbid associations, some authors recommend their systematic search while others do not consider them. Supernumerary nipples do not require any specific treatment. However, surgical excision may be indicated for aesthetic reasons or in case of doubt diagnostic or complications.

**Figure 1:**
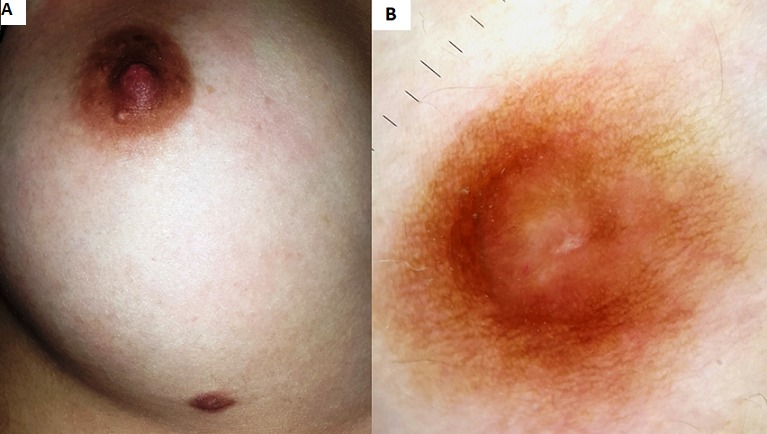
(A) clinical photo: brown plaque localized on the milk line in the underside of the right breast, measuring 1cm, firm in consistency and without any infiltration of the base; (B) dermoscopic photo: peripheral pigmented regular network with a cleft-like appearance in a pigmented homogeneous central area

